# Contractures: Prevalence and Associated Factors in Hospitals and Nursing Homes—Results From Two Consecutive Multicenter Cross‐Sectional Studies

**DOI:** 10.1155/nrp/8458386

**Published:** 2026-10-16

**Authors:** Henriette Elisabeth Neef, Ursula Müller-Werdan, Sandra Strube-Lahmann, Nils Axel Lahmann

**Affiliations:** ^1^ Clinic for Geriatrics and Geriatric Medicine Charité–Universitätsmedizin Berlin Berlin Germany charite.de; ^2^ Stabsstelle Pflegewissenschaft Pflege-und Pflegefunktionsdienst Universitätsmedizin Göttingen Georg-August-Universität Göttingen Germany umg.eu; ^3^ School of Health and Medicine Hochschule für Gesundheit und Medizin MSB Medical School Berlin Berlin Germany

**Keywords:** bedridden, contractures, elderly, hospital, immobility, nursing home, prevalence

## Abstract

**Introduction:**

Contractures frequently may occur in nursing homes and hospitals among predominantly immobile patients and significantly reducing their mobility and quality of life. This study examines the prevalence of contractures among older people in nursing homes and hospitals and shows how often which joint is affected and identifies associated factors.

**Design:**

Two consecutive multicenter cross‐sectional studies (2017 and 2018) involving 2012 individuals aged 65 and older in 12 hospitals and 10 nursing homes.

**Methods:**

Bootstrapping was used to calculate the confidence intervals of the prevalence of contractures in different groups. A multivariable logistic regression model was run comprising all relevant independent associated factors for the presence of contractures.

**Results:**

The overall prevalence of contractures was 11.6% (95% CI 10.2–13.1). In hospitals, the prevalence was 7.7% (95% CI 6.3–9.2), and in nursing homes, it was 19.3% (95% CI 16.3–22.4). Overall, contractures of the upper extremities were more common among nursing home residents (NH) than among hospital patients (H). This pattern was particularly evident for the hands (NH: 30.8 right, 32.9% left vs. H: 21.5% right, 17.7 left), elbows (NH: 21.9 right, 26.0% left vs. H: 10.8% right, 9.5 left), and shoulders (NH: 28.8 right, 29.5% left vs. H: 18.4% right, 14.6 left). For the lower extremities, a more heterogeneous pattern was observed.

**Statistical Analysis:**

The logistic regression model showed that the odds ratio for having at least one contracture for nursing home residents compared with hospital patients was 3.50 (95% CI 2.30–5.19), for age (years) 0.97 (95% CI 0.95–0.99), for urinary incontinence 1.65 (95% CI 1.14–2.38), and for bedridden status 4.15 (95% CI 2.88–5.97).

**Conclusion:**

This study highlights a high prevalence of contractures, particularly in nursing homes compared with hospitals. Identified associated factors such as urinary incontinence and bedridden status may represent potential targets for preventive and nursing interventions, although causal relationships cannot be inferred from this cross‐sectional design. Improved understanding of the prevalence and associated factors of contractures can help enhance quality of care and reduce contracture risk in older, immobile patients, especially in long‐term care settings.

## 1. Introduction

A contracture is a permanent restriction of movement accompanied by loss of function. Lack of movement causes tendons, ligaments, and muscles to shorten, significantly reducing mobility and quality of life. Contractures are commonly categorized as myogenic, atherogenic, or soft‐tissue (scar) contractures [1–4]. Neurological disease and bedridden status may lead to muscle shortening and restricted range of motion (ROM), both passive (PROM) and active. Joint immobilization, fractures, osteochondritis, osteoarthritis, or systemic sclerosis may cause structural changes in bone, cartilage, and joint capsules. Prolonged immobilization in atherogenic contractures may lead to joint ossification and permanent loss of function [5, 6]. Scar contractures arise from connective tissue remodeling during healing after severe burns or large wounds [3]. Older adults, particularly those living in nursing homes or hospitalized for acute or chronic conditions, are at increased risk of developing contractures [5, 7]. Age‐related physiological changes such as sarcopenia, reduced muscle elasticity, decreased collagen turnover, and diminished joint lubrication reduce the musculoskeletal system’s ability to tolerate immobilization and recover from inactivity. In addition, older people are more likely to experience prolonged periods of immobility due to acute illness, multimorbidity, or functional dependency. The prevalence of neurological disorders (e.g., stroke, Parkinson’s disease, and dementia) and chronic musculoskeletal conditions further contributes to limited mobility and abnormal muscle tone, both of which are key risk factors for contracture development. Contractures occur frequently in institutional care settings, including hospitals and nursing homes, where residents and patients are often predominantly immobile or dependent on assistance for movement.

Studies have identified other factors associated with contractures in older people such as unloading/unweighting [4], urinary incontinence [8], diabetes [9], and neurological disease [10]. Once established, contractures can severely restrict the ability to perform activities of daily living, including self‐care, mobility, and personal hygiene, leading to loss of autonomy and increased need for care [6, 11]. Seemingly simple tasks such as toothbrushing or preparing food can present substantial challenges [12, 13]. Fewer contractures enable greater independence, social participation (for example, crafts, painting, and music), and better quality of life [14]. Contractures significantly restrict these possibilities, increasing the risk of depression and social isolation in later life [15]. The existing literature on prevalence is sparse and heterogeneous [16, 17]. Offenbächer et al. noted that “only a minority of studies were performed in a geriatric setting,” limiting interpretation for older populations [4]. The lack of robust data and the potential benefits for prevention and treatment justify further research on contractures [6]. Moreover, prevalence data are often lacking or inconsistent across different countries and healthcare systems, making it difficult to estimate the true extent of the problem and to develop targeted prevention and intervention strategies.

A clearer understanding of the prevalence of contractures and associated risk factors in older patients is essential to raise awareness of this often‐underestimated condition, to support early detection and preventive measures, and ultimately to improve quality of care and quality of life for older adults.

This study aims to determine the prevalence of contractures in hospitals and nursing homes among patients aged over 65 and to identify factors associated with increased contracture risk. Research questions: • What is the prevalence of contractures among older patients in hospitals and among residents of nursing homes? How often do they occur in individuals affected and which joints are predominant? Which individual factors are associated with contractures?

## 2. Materials and Methods

### 2.1. Study Design

In each of two consecutive years (2017 and 2018), a multicenter (12 hospitals and 10 nursing homes) cross‐sectional point‐prevalence study in Germany was conducted.

### 2.2. Data Collection

The data‐collection method was identical in both years, and surveys were conducted by trained nursing staff. Data on prevalence, localization, and potential associated factors for contractures were collected from older patients in hospitals and residents in nursing homes using written survey forms (Supporting 3). Participating facilities received the necessary materials. Collected data were machine‐read, then checked and prepared for analysis.

### 2.3. Study Participants

Only individuals aged 65 years and older were included. Informed consent was obtained from all residents and patients who had capacity, or from next of kin where appropriate. No other exclusion criteria were applied.

### 2.4. Outcome Measures

During hospital or nursing home stay, demographic variables such as age, sex, and BMI were recorded. Medical records were checked for diabetes mellitus, stroke, and dementia. Apart from very well‐established associations like bedridden status and stroke, we included some other factors such as dementia, diabetes, and urinary incontinence, as well as age and BMI for exploratory purposes. Bedridden status was recorded using the “mobility” and “activity” items of the Braden scale for pressure ulcer risk [18]. On the Braden Scale [19], the mobility section describes the extent to which a person is able to change and control their body position in bed or a chair independently. It assesses whether changing position is possible independently, with assistance, or not at all. The outcome measure “bedridden” stands for immobility. Urinary incontinence was recorded using the ICIQ scale [20, 21]. The ICIQ assesses urinary incontinence using a standardized questionnaire. It records the frequency of urine leakage, the amount of urine leakage, and the impact on everyday life. Data on care setting (hospital or nursing home) were also collected.

### 2.5. Localization

Descriptive statistical analyses were conducted to determine the prevalence of contractures at each examined joint separately for hospital patients and nursing home residents. For each joint, the proportion of individuals presenting with a contracture was calculated. All analyses were performed separately for the left and right sides where applicable (e.g., hands, elbows, shoulders, knees, feet, and hips). Results are presented as percentages with corresponding 95% confidence intervals.

### 2.6. Bias

Social desirability bias refers to the tendency of participants or institutions to provide responses or data that are perceived as more acceptable or favorable rather than fully accurate. In the context of this study, staff in hospitals and nursing homes may have underreported the presence of contractures or overreported preventive practices in order to present their institution in a more positive light. To reduce this potential bias, participating institutions were pseudonymized, thereby minimizing concerns about reputational consequences and encouraging more honest reporting.

To minimize selection bias and ensure validity, as many individuals as possible were included. When persons lacking capacity to consent, next of kin gave consent to capture a broad cross section of the population aged over 65. Data were collected by trained staff using standard procedures to limit information bias. Potential associated factors were assessed in a multivariable logistic regression to reduce confounding. To reduce nonresponse bias, the questionnaire was kept simple and brief [22].

### 2.7. Study Size

As the study was primarily exploratory, no a priori sample size calculation was performed. However, the achieved sample size may be considered sufficient to detect clinical relevant differences in groups and yields relatively precise estimates and narrow confidence intervals of, i.e., prevalence figures [23].

### 2.8. Statistical Methods

For categorical variables, absolute and relative frequencies were used. For metric data—where the assumption of normal distribution was not seriously violated—means and standard deviations were calculated. Bootstrapping with 500 repetitions was used to calculate 95% confidence intervals for categorical variables. To assess the robustness of this choice, the bootstrap procedure was additionally repeated with 1000 (the default setting in SPSS) and 2000 repetitions; the resulting confidence intervals did not differ from those obtained with 500 repetitions at the first decimal place, confirming that the reported intervals are stable and not sensitive to the chosen number of repetitions. Univariate analyses between potential associated factors and contractures were performed; chi‐square tests were used for proportions and *t*‐tests for metric variables (age and BMI). A multivariable logistic regression model was run comprising all relevant independent associated factors for the presence of contractures. For all inferential statistics, an alpha of < 0.05 (two‐sided) was considered statistically significant. Two‐way interaction terms between selected clinical variables (dementia x stroke, dementia x diabetes mellitus, diabetes mellitus x stroke, and bedridden status x stroke) were additionally tested in the year‐stratified regression models; none reached statistical significance (see Supporting Tables S1 and S2).

### 2.9. Ethical Considerations

This study was approved by the ethics committee of the research group at the university under approval number (EA/272/00). All participants provided informed consent in accordance with the Declaration of Helsinki.

## 3. Results

### 3.1. Sample

Of the total sample, 680 individuals (33.8%) were from nursing homes and 1332 individuals (66.2%) were hospital patients. Among hospital patients, 631 (47.4%) were recruited in 2017 and 701 (52.6%) in 2018 (Table 1). In nursing homes, 283 patients (41.6%) were included in 2017 and 397 (58.4%) in 2018. In 2017, patient participation across hospitals ranged from 53 to 162 individuals (2018: 40 to 347 patients). Within nursing homes, the number of participating residents ranged from 5 to 142 (2018: 24 to 147 residents); the facility with only 5 participants was a specialized nursing care institution, which likely accounts for its comparatively small resident population. The discrepancy between the figures in the text and those in Table 2 is due to missing data for 20 people in the hospital and 13 people in the nursing home. No information was provided as to whether these individuals have contractures or not. Sex was available for 1966 patients: 1131 female patients (57.5%) and 835 male patients (42.5%) of valid cases. Diabetes mellitus was present in 556 patients (27.6%), stroke in 302 patients (15%) of the total sample, and 468 patients with dementia (23.3%). The mean age was 80.9 years, and the mean BMI was 25.7 kg/m^2^.

**TABLE 1:** Patients in 2017 and 2018.

Institution	Year	*n*	%
Hospital	2017	631	47.4
	2018	701	52.6
	Total	1332	100.0

Nursing home	2017	283	41.6
	2018	397	58.4
	Total	680	100.0

**TABLE 2:** Prevalence of contractures in hospitals and nursing homes.

Type	Contracture	Frequency	Percent	Lower 95%	Upper 95%
Hospital	no	1211	92.3	90.8	93.7
	yes	101	7.7	6.3	9.2

Nursing home	no	538	80.7	77.6	83.7
	yes	129	19.3	16.3	22.4

### 3.2. Prevalence

Prevalence among all individuals was 11.6% (95% CI 10.2–13.1). Of 2012 patients, 1979 stated whether they had a contracture; 33 patients did not provide this information. Table 2 shows prevalence differences between hospitals and nursing homes.

One hundred and one hospital patients had at least one contracture (prevalence 7.7%, 95% CI 6.3–9.2). In nursing homes, prevalence was 19.3% (95% CI 16.3–22.4). The number of contractures per individual affected ranged from 1 to 13 in hospitals and 1 to 12 in nursing homes, reflecting an average of 2.3 (sd 2.1) in prevalent hospital patients and 3.3 (sd 2.3) in nursing home residents. This difference in groups was statistically significant.

The frequency of contractures varied between hospitals and nursing homes and across the examined joints. Overall, contractures of the upper extremities were more common in nursing home residents than in hospital patients. For the hands, frequency in the hospital setting was 17.7% (95% CI: 11.7–23.6) on the left and 21.5% (95% CI: 15.3–27.9) on the right, compared with 32.9% (95% CI: 25.4–41.0) and 30.8% (95% CI: 23.2–38.6), respectively, in nursing homes. Similar differences were observed for the elbows: 9.5% (95% CI: 5.1–14.7) left and 10.8% (95% CI: 6.3–16.0) right in hospitals, 26.0% (95% CI: 19.2–33.8) left and 21.9% (95% CI: 15.2–28.9) right in nursing homes. 14.6% [95% CI: 9.3–20.7] of the patients in hospitals show contractures in the left shoulder and 18.4% (95% CI: 12.4–24.5) right; nursing homes: 29.5% (95% CI: 21.6–36.8) left and 28.8% (95% CI: 21.7–36.4) right.

For the lower extremities, a more heterogeneous pattern was observed. Knee contractures were more prevalent in nursing home residents (left: 37.7% [95% CI: 30.1–46.3]; right: 39.0% [95% CI: 30.7–47.1]) than in hospital patients (left: 29.1% [95% CI: 21.8–36.3]; right: 27.8% [95% CI: 21.1–34.2]). In contrast, the frequency of hip contractures was comparable between settings (H: 14.6% [95% CI: 9.5–20.4] left and 15.8% [95% CI: 10.3–22.0] right; nursing home: 13.7% [95% CI: 8.4–19.6] left and 16.4% [95% CI: 10.5–22.8] right). Similarly, foot contractures showed comparable frequency in hospitals (left: 20.9% [95% CI: 14.6–27.9]; right: 22.2% [95% CI: 16.1–29.1]) and nursing homes (left: 20.5% [95% CI: 14.4–28.1]; right: 17.1% [95% CI: 11.0–23.5]). Neck contractures were observed in 12.0% (95% CI: 7.1–17.6) of hospital patients and 17.1% (95% CI: 11.4–23.2) of nursing home residents. The localization and frequency of contractures across all examined joints, stratified by care setting, are summarized in Figure 1.

**FIGURE 1:**
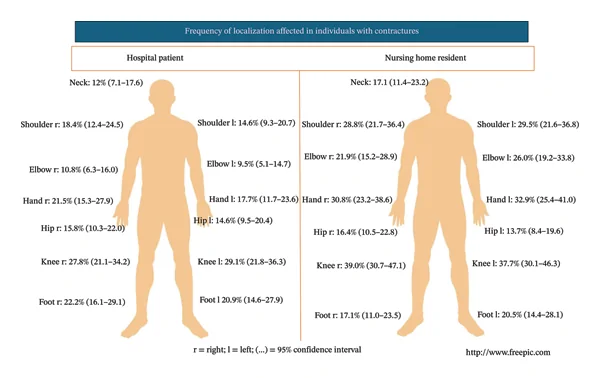
Localization of contractures.

### 3.3. Associated Factors

Boxplots were created for age (Figure 2) and BMI (Figure 3).

**FIGURE 2:**
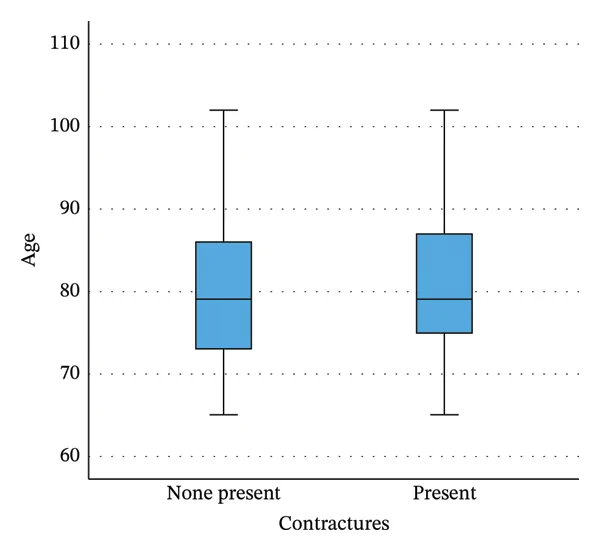
Age and contractures.

**FIGURE 3:**
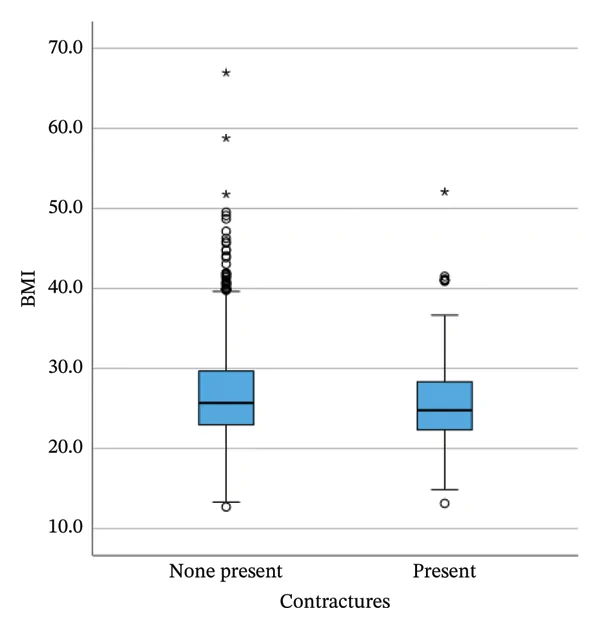
BMI and contractures.

According to Figure 2, individuals without contractures had a mean age of 79.6 years (median 79; interquartile range 73–86). Patients with a contracture had a mean age of 80.9 years; none were younger than 75. Notably, while patients with contractures were older on average than those without (80.9 vs. 79.6 years), age yielded an odds ratio below 1.0 in the multivariable model (OR 0.97, 95% CI 0.95–0.99; *p* = 0.008). This apparent reversal is likely attributable to confounding by variables strongly correlated with age, particularly nursing home residence and bedridden status, both of which were independently associated with contractures and are more prevalent in older individuals.

According to Figure 3, BMI in the no‐contracture group ranged from 13 to 40 kg/m^2^, with outliers at the higher end; the median BMI was 26 kg/m^2^, 25% were < 23 kg/m^2^ and 25% were > 30 kg/m^2^. Patients with contractures showed lower BMI values (first quartile 22 kg/m^2^, median 25 kg/m^2^, and third quartile 28 kg/m^2^). Six categorical associated factors were predefined: urinary incontinence, bedriddenness, dementia, stroke, diabetes, and sex. Patients were classified as present/absent for each factor. Figure 4 shows relative prevalences and CIs for each group.

**FIGURE 4:**
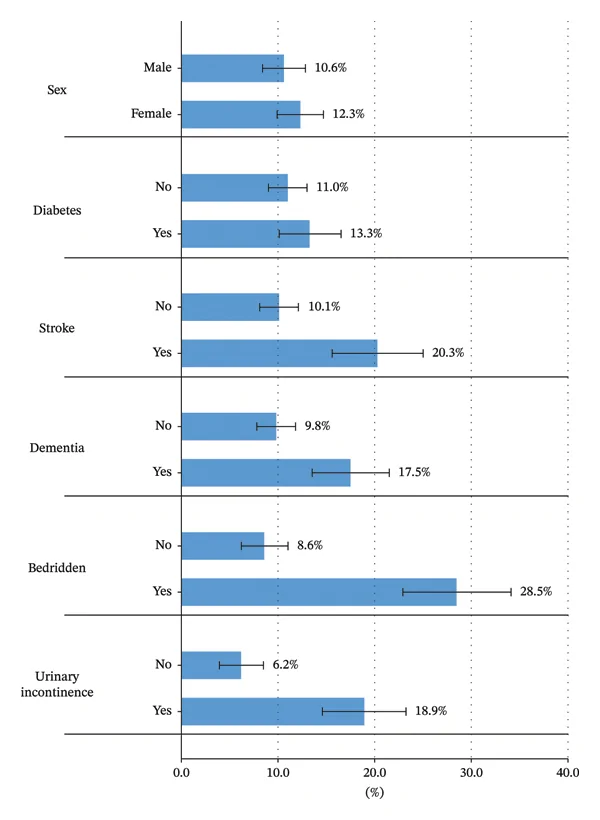
Possible associated factors for contractures.

Eight hundred and thirty‐eight individuals had urinary incontinence and 288 were bedridden. Contracture status was available for 547 of the 556 individuals with diabetes (98.4%); of these, 13.3% had a contracture. Among patients with stroke, 20.3% had a contracture. 17.5% of 468 individuals with dementia had a contracture. Among bedridden individuals, 28.5% had a contracture. In 18.9% of individuals with urinary incontinence, at least one contracture was present.

### 3.4. Multivariable Regression Model

In Table 3, all associated factors and care setting (nursing home coded “yes” vs. hospital coded “no”) were entered into a logistic regression model for the presence of at least one contracture.

**TABLE 3:** Multivariable logistic regression model.

Variable	Odds ratio	*p.*	Under 95% CI	Over 95% CI
Setting (nursing home = 1)	3.50	< 0.001	2.3	5.19
Sex (female = 1)	1.00	1.00	0.71	1.41
BMI (kg/m^2^)	0.97	0.08	0.95	1.00
Age (years)	0.97	0.008	0.95	0.99
Urinary incontinence (present = 1)	1.65	0.008	1.14	2.38
Bedridden (present = 1)	4.15	< 0.001	2.88	5.97
Diabetes m. (present = 1)	1.17	0.36	0.83	1.66
Stroke (present = 1)	1.33	0.15	0.91	1.96
Dementia (present = 1)	0.95	0.81	0.64	1.42

*Note:* Model fit: *n* = 1673; −2 log‐likelihood = 1066.07; Cox &Snell *R* ^2^ = 0.082; Nagelkerke *R* ^2^ = 0.159.

According to Table 3, age, urinary incontinence, bedridden status, and residence in a nursing home were statistically significant. Odds ratios were 3.50 (95% CI 2.30–5.19) for nursing home residents, 0.97 (95% CI 0.95–0.99) for age (years), 1.65 (95% CI 1.14–2.38) for urinary incontinence, and 4.15 (95% CI 2.88–5.97) for bedridden status. Other factors were not statistically significant.

## 4. Discussion

This study examined the contracture prevalence in individuals aged over 65 in nursing homes and hospitals. Overall, 11.6% of participants had at least one contracture. Despite their prevalence, research on contractures remains relatively limited [4]. This study summarizes current knowledge and provides new insights. It is important to acknowledge that, to the authors’ knowledge, more recent multicenter prevalence data for Germany or comparable European settings are currently unavailable. Therefore, despite the 2017‐2018 data collection window, this study represents the best available evidence on contracture prevalence in this population. The prevalence and frequency of contractures was considerably higher among nursing home residents than among hospital patients, indicating that care setting may represent an important environmental factor for contracture risk. Other factors associated with contractures included bedridden status, urinary incontinence, and higher age.

### 4.1. Nursing Homes

The higher prevalence in nursing homes may reflect multiple factors: Residents of long‐term care facilities tend to be older and are more likely to be permanently dependent on assistance with activities of daily living, which often leads to prolonged physical inactivity [24, 25]. Lack of mobilization is a probable contributor. Time and staff shortages can limit opportunities for movement [26]; immobile patients require more nursing time, increasing workload and potentially creating a vicious cycle of increasing immobility and contracture risk. Studies from England (1996) and Philadelphia (2010) reported contracture prevalences of 55% and 61.2%, respectively, in care facilities [8, 27], supporting the association between nursing home residence and higher contracture risk. In contrast, hospitals generally provide more frequent patient mobilization and there may be a greater access to physiotherapy and rehabilitation services.

### 4.2. Age

Age emerged as an associated factor in the univariate analysis; patients with contractures were on average approximately 1 year older than those without, and all patients with contractures were at least 75 years old. However, in the multivariable model (Table 2), age appeared as a protective factor (OR 0.97, 95% CI 0.95–0.99), which seems counterintuitive at first glance. This reversal most likely reflects confounding rather than a true biological effect: Older age is strongly associated with nursing home residence and bedridden status, both of which were the dominant independent predictors of contracture in the multivariable model. Once these variables are adjusted for, the residual effect of age reverses direction—a phenomenon consistent with Simpson’s paradox. Age should therefore not be interpreted as an independent risk factor for contractures; its effect appears to be largely mediated through functional status and care setting. From a biological perspective, aging reduces regenerative capacity and muscle mass [28], which may impair the ability to compensate for immobility—suggesting that age likely acts on contracture risk indirectly through these functional pathways rather than as a direct independent determinant.

### 4.3. Bedridden Status

Nearly one in six participants in the present study was bedridden, and almost one‐third of these had at least one contracture. Age and bedridden status are correlated: Degenerative and chronic diseases increase with age, while muscle and bone mass decline, increasing contracture risk [5, 28, 29]. Passive movements by staff or physiotherapists do not fully substitute for active muscle use. Dittmer et al. reported that a muscle not used for 1 week loses 10%–15% strength [30]. Thus, bedridden status is a clear risk factor. As stroke and bedridden status are clinically related, we examined their association to rule out collinearity; the phi coefficient was 0.153 in hospitals and 0.258 in nursing homes, indicating weak collinearity in both settings, which does not preclude the interpretation of both variables in the multivariable model. The comparatively weaker and nonsignificant association of stroke with contractures in the multivariable model, despite a significant univariate association, is therefore more likely explained by mediation through bedridden status and care setting than by collinearity.

### 4.4. Urinary Incontinence

Limited mobility and urinary incontinence are linked [31]. Many bedridden patients are also incontinent, possibly because they cannot reach the toilet independently. A Japanese study reported urinary incontinence prevalence of over two‐thirds in patients aged over 70 [32], indicating associations among age, incontinence, and immobility. Age, urinary incontinence, and immobility are important associated factors for contracture development.

### 4.5. Other Factors

Current evidence suggests that contractures represent a common and clinically relevant complication in patients with neurological disorders, particularly following stroke [33]. The development of contractures is closely associated with central nervous system injury affecting motor pathways. Damage to corticospinal and related motor networks leads to muscle weakness, impaired motor control, and frequently altered muscle tone, often manifesting as spasticity. These changes disrupt the balanced activation of agonist–antagonist muscle groups and promote abnormal movement patterns and limb postures, ultimately reducing active joint movement and functional use of the affected limb [34].

Notably, in our analyses, stroke exhibited a strong association with the presence of contractures in bivariate analysis; however, this association did not retain statistical significance in multivariable models, indicating that its effect may be mediated by other clinical factors. However, spasticity alone does not fully explain the development of contractures. Increasing evidence indicates that reduced mobility and prolonged immobilization play a central role in their pathophysiology. Limited joint movement decreases the regular mechanical stretching of muscles and periarticular tissues, which over time promotes fibrotic remodeling and structural shortening of these tissues, resulting in persistent restriction of joint mobility. Additional factors such as pain, sensory impairments, and cognitive deficits [35] may further exacerbate immobilization and thereby increase the risk of contracture formation.

Overall, contractures in neurological disorders should be considered a multifactorial consequence of central nervous system injury, with reduced mobility representing a key mechanism driving progressive structural changes in musculoskeletal tissues.

Taken together, these results suggest that the association between neurological diagnoses and contractures may be mediated by immobility and care setting, rather than reflecting a direct, independent effect.

### 4.6. Localization

In addition to the identified associated factors, the distribution of contractures differed between anatomical locations. Contractures of the upper extremities, particularly of the hands, elbows, and shoulders [17], were more frequent in nursing home residents than in hospital patients. Knee contractures also occurred more often in nursing home residents.

In contrast, the frequency of hip and foot contractures was similar in both care settings, suggesting that these joints are influenced by factors present in both hospitals and nursing homes. Neck contractures were observed slightly more often among nursing home residents.

Overall, these findings indicate that the anatomical distribution of contractures is not uniform but varies by care setting and joint. This underlines the need for joint‐specific prevention strategies, with particular attention to upper extremity and knee joints in long‐term care.

### 4.7. Temporal Heterogeneity Between Survey Years

A comparison between the two survey years revealed a notable shift in the setting‐specific prevalence of contractures. In 2017, the difference in prevalence between hospitals and nursing homes was not statistically significant (9.3% vs. 12.5%, *p* = 0.150), whereas in 2018, this difference was pronounced and highly significant (6.3% vs. 24.4%, *p* < 0.001). This pattern is also reflected in the year‐stratified multivariable models (Supporting Tables S1 and S2): In 2017, bedridden status and care setting were the only significant predictors of contracture, whereas in 2018, care setting showed a markedly stronger association, and age and urinary incontinence additionally reached significance.

Several factors may plausibly explain this temporal shift. First, the 2018 sample included a larger proportion of nursing home residents relative to hospital patients than the 2017 sample, and case‐mix (e.g., functional dependency and frailty) may have differed between the two cohorts independent of setting. Second, one hospital contributed a disproportionate share (26.1%) of the 2018 sample, and if this facility systematically differed in patient acuity, screening practices, or documentation standards, it could have influenced the pooled hospital estimate for that year. Third, unmeasured changes in staffing, mobilization practices, or admission criteria between the two survey years cannot be excluded given the cross‐sectional, nonlongitudinal design.

Because the pooled model presented in Table 3 combines both survey years, it does not capture this temporal heterogeneity. We therefore report the year‐stratified regression results separately in the Supporting Information (Tables S1 and S2) and recommend that readers interpret the pooled odds ratios as an average association across the two survey years rather than as a temporally stable effect. Related to this issue, individuals in this study were nested within 22 participating facilities, and one hospital alone accounted for 26.1% of the 2018 sample. This clustering was not formally accounted for in the logistic regression model (e.g., via a facility‐level random intercept or generalized estimating equations), which may result in underestimated standard errors and overly narrow confidence intervals. We acknowledge this as an additional limitation of the present analysis and recommend that future studies of contracture prevalence in multicenter samples account for facility‐level clustering explicitly.

### 4.8. Limitations

The study gives insight into contracture prevalence and associated factors but has limitations. Voluntary participation and pseudonymization of institutions could introduce selection bias; facilities under staff pressure may have declined participation despite caring for many patients with contractures. Prior studies using similar methods indicate that the sample represents the target populations well. Information bias may arise from varying experience among investigators [36, 37], but clear definitions were provided to reduce assessment bias. Contracture assessments were performed by fully trained German registered nurses (*Pflegefachkräfte*); however, the absence of standardized goniometric thresholds and interrater reliability coefficients means that measurement bias cannot be fully excluded, as individual clinical thresholds may have varied between assessors and facilities, potentially leading to under‐ or overreporting of contracture prevalence. Furthermore, the study did not perform a formal mediation analysis to disentangle the interrelated effects of age, care setting, and functional status on contracture prevalence. The observed reversal of the age effect between univariate and multivariable analyses suggests potential mediation or confounding that warrants further investigation in future studies. Due to its cross‐sectional design, this study cannot establish causal or temporal relationships between associated factors and contractures. The study did not include detailed measures of mobility or neuromuscular function (e.g., spasticity or functional status), which may have affected the interpretation of associated factors.

Care practices have evolved considerably in the years since data collection, particularly in the context of the COVID‐19 pandemic, which profoundly affected both hospital and nursing home environments. Restricted mobility, isolation measures, and disruptions to rehabilitative care during the pandemic may have further increased the risk of contracture development in vulnerable older adults. Against this backdrop, the present findings should be interpreted as a prepandemic baseline, underscoring the urgent need for updated, contemporary surveillance data to capture potential shifts in contracture prevalence in the postpandemic period.

Despite these limitations, the findings are relevant and should be interpreted in context.

## 5. Conclusion

The prevalence of contractures in this study was substantial, with 11.6% of individuals aged 65 years and older affected. Compared with previous reports from hospital and long‐term care settings, this prevalence indicates that contractures remain a clinically relevant and frequently occurring condition in older populations. Contracture development appears to be multifactorial, with bedridden status, urinary incontinence, and nursing home residence emerging as robust associated factors; the apparent protective association with age likely reflects confounding by care setting and functional status rather than a direct effect (see Section 4.2). In addition, differences in the anatomical distribution of contractures between care settings were observed, with higher frequency particularly in the upper extremities and knees among nursing home residents. These results should aid early detection and prevention. Given ongoing demographic change and an aging population, contractures warrant greater attention to preserve autonomy and quality of life. Early and repeated screening for restriction in ROM, particularly in older and immobile persons, is essential to enable timely identification of contractures and to initiate preventive or therapeutic measures. Promoting and maintaining mobility should therefore be regarded as a central component of high‐quality care for older adults in both hospitals and nursing homes. In line with the cross‐sectional design of the study, these associations do not imply causal relationships.

## 6. Implications for Practice and Research

Preventing bedridden status is a key preventive strategy, as mobility supports independence in daily activities and overall well‐being. Screening for ROM restrictions should be considered as age increases. Preventing bedridden status requires more frequent intervention by nursing staff, physiotherapists, and occupational therapists. Staff shortages in hospitals and nursing homes will increase the care gap; preventive work will become ever more important. Digital tools, such as step counters in smartphones and smartwatches, could monitor movement and detect declines in physical activity; data could be reviewed during primary care visits. Inpatient care should prioritize early mobilization and movement therapy to reduce immobility and consequent workload. Future research should focus on developing and evaluating standardized approaches for detection, documentation, and treatment of contractures in outpatient and inpatient care. Systematic documentation should include contracture location and degree to monitor progression and tailor treatment.

## Funding

Funding was provided by the research organization only.

Open access funding was enabled and organized by Projekt DEAL.

## Conflicts of Interest

The authors declare no conflicts of interest.

## Supporting Information

Additional supporting information can be found online in the Supporting Information section.

## Supporting information


**Supporting Information** Three supporting files accompany this manuscript. Supporting 1 (Table S1) and Supporting 2 (Table S2) present the year‐stratified multivariable logistic regression models for 2017 and 2018, respectively, including the tested two‐way interaction terms and corresponding model‐fit statistics, together with a short explanatory summary. Supporting three is the written survey questionnaire used by trained nursing staff for data collection in participating hospitals and nursing homes. These materials are provided to support transparency and reproducibility of the reported analyses.

## Data Availability

Datasets used and/or analyzed during the current study are available from the corresponding author on reasonable request.
