# Supplementary material for: Contractures: Prevalence and Associated Factors in Hospitals and Nursing Homes—Results From Two Consecutive Multicenter Cross‐Sectional Studies
Source: Nurs Res Pract. 2026 Oct 16;2026:8458386. doi: 10.1155/nrp/8458386 (PMC13673220; doi:10.1155/nrp/8458386)
Supplement: Supplementary file 1 [file NRP-2026-8458386-s001.docx]

Supplements

Logistic regression analyses of predictors of contracture prevalence, stratified by year (2017 and 2018) and excerpt of Original Questionnaire (Original in German, containing all relevant information)

Supplement S1 Logistic Regression on Contracture (present = 1)

| Year 2017 (n = 742) | | | | | |
| --- | --- | --- | --- | --- | --- |
|  | | | | p-value | Exp(B) |
|  | setting (nursing home =1) | | | .032 | 1.985 |
|  | sex (female =1) | | | .483 | .830 |
|  | BMI (kg/m2) | | | .286 | .973 |
|  | age (years) | | | .298 | .981 |
|  | bedridden(present=1) | | | <.001 | 5.844 |
|  | stroke (present=1) | | | .917 | 1.054 |
|  | diabetes m. (present=1) | | | .845 | 1.074 |
|  | dementia (present=1) | | | .606 | .801 |
|  | stroke (present=1) | | | .344 | 1.797 |
|  | dementia (present=1) by diabetes m. (present=1) | | | .637 | 1.320 |
|  | stroke (present=1) | | | .592 | 1.390 |
|  | urinary incontinence (present=1) | | | .289 | 1.365 |
|  | Bedridden (present=1) by stroke (present=1) | | | .601 | .733 |
|  | constant | | | .755 | .599 |
| a. year= 2017 | | | | | |
| b. Variables entered in Step 1: Type2, Gender, BMI, age, Is the resident/patient bedridden?, Stroke, Diabetes mellitus, Dementia, Dementia * Stroke, Dementia * Diabetes mellitus, Diabetes mellitus * Stroke, Urinary incontinence, Is the resident/patient bedridden? * Stroke. | | | | | |
| Supplement S1a Table of Model Summary for Logistic Regression S1 | | | | | |
| step | | -2 Log-Likelihood | Cox & Snell R-Square | Nagelkerkes R-Square | |
| 1 | | 449.613^b^ | .070 | .141 | |
| a. year = 2017 | | | | | |
| b Estimation stopped at iteration number 5 because the parameter estimates changed by less than .001. | | | | | |

Supplement S2 Logistic Regression on Contracture (present = 1
Year 2018 (n = 890)

|  | | p-value | | Exp(B) |
| --- | --- | --- | --- | --- |
|  | setting (nursing home =1) | | <.001 | 5.230 |
|  | sex (female =1) | | .555 | 1.151 |
|  | BMI (kg/m2) | | .386 | .982 |
|  | age (years) | | .007 | .958 |
|  | bedridden(present=1) | | <.001 | 3.241 |
|  | stroke (present=1) | | .452 | 1.395 |
|  | diabetes m. (present=1) | | .499 | 1.237 |
|  | dementia (present=1) | | .633 | .855 |
|  | stroke (present=1) | | .985 | .990 |
|  | dementia (present=1) by diabetes m. (present=1) | | .746 | 1.169 |
|  | stroke (present=1) | | .438 | .643 |
|  | urinary incontinence (present=1) | | .025 | 1.763 |
|  | Bedridden (present=1) by stroke (present=1) | | .543 | 1.409 |
|  | constant | | .789 | 1.444 |
| a. year = 2018 | | | | |
| b. Variables entered in Step 1: Type2, Gender, BMI, age, Is the resident/patient bedridden?, Stroke, Diabetes mellitus, Dementia, Dementia * Stroke, Dementia * Diabetes mellitus, Diabetes mellitus * Stroke, Urinary incontinence, Is the resident/patient bedridden? * Stroke. | | | | |

| Supplement S2a Table of Model Summary for Logistic Regression S2 | | | |  |
| --- | --- | --- | --- | --- |
| **Model Summary** | | | | |
| step | -2 Log-Likelihood | Cox & Snell R-Square | Nagelkerkes R-Square | |
| 1 | 598.55^b^ | .106 | .199 | |
| a. year = 2018 | | | | |
| b. Estimation stopped at iteration number 5 because the parameter estimates changed by less than .001. | | | | |

Explanation of the Regression Tables

To account for potential temporal variation, separate logistic regression analyses were conducted for the years 2017 (n = 742) and 2018 (n = 890) to identify predictors of contracture prevalence, including several two-way interaction terms. According to Table S1, in 2017, being bedridden was the strongest predictor of contracture prevalence (Exp(B) = 5.84, p < .001), alongside the nursing home setting (Exp(B) = 1.99, p = .032); no other variables or interaction terms reached significance. In 2018, being bedridden remained a highly significant predictor, with a slightly smaller effect size (Exp(B) = 3.24, p < .001), while nursing home setting showed an even stronger association with contracture prevalence (Exp(B) = 5.23, p < .001). Additionally, age (Exp(B) = 0.96, p = .007) and urinary incontinence (Exp(B) = 1.76, p = .025) emerged as significant predictors in 2018, whereas they had not been significant in 2017. None of the tested interaction terms (e.g., dementia × stroke, diabetes × stroke) reached statistical significance in either year. Model fit was modest in both years, with Nagelkerke's R² indicating that the models explained 14.1% (Tables S1a) of variance in contracture prevalence in 2017 and according to table S2a 19.9% in 2018 (Cox & Snell R² = .070 and .106, respectively), suggesting that unmeasured factors likely contribute substantially to contracture development. Taken together, these findings indicate that immobility (being bedridden) constitutes a robust and consistent risk factor for contractures across both years, while the relevance of age and urinary incontinence appears to have increased over time, pointing to a possible shift in the relevant risk profile.

Supplement S3 Original Questionnaire (Original in German, containing all relevant information)
